# Quantitative Proteomic Analysis Reveals the Sites Related to Acetylation and Mechanism of ACY-1215 in Acute Liver Failure Mice

**DOI:** 10.3389/fphar.2019.00653

**Published:** 2019-06-11

**Authors:** Wen-bin Zhang, Hai-yue Zhang, Yao Wang, Fang-zhou Jiao, Lu-wen Wang, Zuo-jiong Gong

**Affiliations:** Department of Infectious Diseases, Renmin Hospital of Wuhan University, Wuhan, China

**Keywords:** ACY-1215, histone deacetylase 6, acute liver failure, necrosis, quantitative proteomic analysis

## Abstract

**Background:** ACY-1215 is a well-known selective histone deacetylase 6 (HDAC6) inhibitor, and it has been considered as a potential therapeutic drug in inflammatory diseases, including acute liver failure (ALF). However, little is known about the impact of ACY-1215 treatment on histone lysine acetylation and proteome in ALF. In this study, we aim to investigate whether ACY-1215 has inhibitory effects and mechanism on the necrosis of hepatocytes; moreover, the impact of ACY-1215 treatment on histone lysine acetylation still needs further elucidation.

**Methods:** Male C57/BL6 mice were divided into normal, model, and ACY-1215 groups. ACY-1215 (25 mg/kg) and same amounts of saline were injected intraperitoneally to the mice before the establishment of ALF model induced by lipopolysaccharide (LPS) (100 µg/kg) combined with D-gal (400 mg/kg). All animals were sacrificed after 24 h. In this study, detection programs, including quantitative proteomic analysis, transmission electron microscopy (TEM) micrographs, pathological staining, protein expression, the detection of reactive oxygen species (ROS) as well as glutamic oxaloacetic transaminase (GOT) and glutamic pyruvic transaminase (GPT) measurement.

**Results:** The function of liver and the necrosis of hepatocytes in ALF mice were significantly normalized by ACY-1215 pretreatment. The quantitative proteomic analysis revealed that ACY-1215-restrained oxidative phosphorylation normalized the function respiratory electron-transport chain in the mitochondria. Moreover, pretreatment of ACY-1215 not only normalized the structure of mitochondria but also inhibited the generation of reactive oxygen species (ROS).

**Conclusions:** ACY-1215 was able to inhibit necrosis of hepatocytes in ALF mice through regulating the mitochondrial-mediated oxidative stress, and we identified the common sites related to acetylation level.

## Introduction

Acute liver failure (ALF) is a severe clinical syndrome with significant mortality, which is caused by rapid and extensive necrosis of hepatocytes and loss of hepatocyte function (Zhang et al., 2018c; Rabinowich et al., 2016). Liver transplantation is still the most effective treatment at present, but this application is limited by donor numbers, post-operative complications, and rejection of allogeneic transplants (Jiang et al., 2019; Wang et al., 2019). Therefore, new and effective therapies are urgent needed for ALF.

Lipopolysaccharide (LPS) and d-galactosamine (d-GalN) are commonly used as the experimental ALF animal model which can precisely reflect human liver failure (Kim and Lee, 2013). Endotoxemia and hepatic toxic substances play important roles in the occurrence and development of liver failure, and in LPS/d-GalN-induced ALF model, d-GalN plays a hepatotoxic role, whereas LPS-induced inflammatory response can also aggravate the injury of hepatocytes and further aggravate liver failure (Zong et al., 2014; Bi et al., 2017).

Our previous studies have shown that broad-spectrum histone deacetylase inhibitors (HDACi) had a protective effect on many diseases, including ALF, acute-on-chronic liver failure, and acute kidney injury (Zhang et al., 2015; Zhang et al., 2016; Zhang et al., 2018a). However, nonselective HDAC inhibition may lead to potentially toxic and side effects due to the extensive acetylation of intracellular molecules. Therefore, selective HDACis may be safer and have lower side effects than nonselective HDACis (Prince et al., 2009; Cheng et al., 2019).

Our previous study had reported that the pretreatment of ACY-1215, a leading HDAC6 inhibitor, had a significant inhibitory effect on inﬂammatory responses in ALF mice (Zhang et al., 2018b). However, the effect and underlying mechanisms of ACY-1215 in the necrosis of hepatocytes remains unclear, and the impact of ACY-1215 treatment on histone lysine acetylation and proteome still need further elucidation. So, in the present study, we performed quantitative whole proteome analysis and histone lysine acetylation profile in ALF mice with ACY-1215 pretreatment, which may deepen our understanding of ACY-1215-dependent ALF therapy.

## Materials and Methods

### Animals and Animal Models

All animal experiments were performed in accordance with the institutional guidelines of the Animal Care and Use Committee of Renmin Hospital of Wuhan University and the Guide for the Care of Laboratory Animals published by the US National Institutes of Health (NIH publication no. 85-23, revised 1996). Adult-specific pathogen-free (SPF) male C57BL/6 mice (8–10 weeks old, 18–22 g) were purchased from the Laboratory Animal Science, Chinese Academy of Medical Sciences (CAMS) & Peking Union Medical College (PUMC) (Beijing, China). All animals were allowed to acclimatize to the laboratory environment for 7 days. Forty-five mice were randomly divided into normal group (15 mice), model group (15 mice), and ACY-1215 group (15 mice). The ALF models were induced by intraperitoneal injection of LPS (100 μg/kg, L2880; Sigma) and D-gal (400 mg/kg, G0050; Sigma) as described previously (Zhang et al., 2016). High dose of ACY-1215 (25 mg/kg, S8001, Selleck) were given to the ACY-1215 group 2 h before ALF. Meanwhile, the mice in normal group and model group received the same volume of normal saline. All animals were sacrificed in 24 h.

### Histological Examination

The livers were excised, washed with phosphate buffer saline (PBS) and fixed overnight by the 10% formalin. Then, the liver tissues were embedded in paraffin, cut into 4- to 5-μm-thick slide sections. Hematoxylin & eosin (HE) staining were for histological analysis, and the pathological changes were evaluated under light microscope. Terminal deoxynucleotidyl transferase (TdT)-mediated 2’-Deoxyuridine 5’-Triphosphate (dUTP) Nick-End Labeling (TUNEL) were used to observe the necrosis of hepatocytes.

### Glutamic Oxaloacetic Transaminase/Glutamate Pyruvic Transaminase Measurement

To collect the supernatant, all liver tissue was homogenized with homogenizer, then all samples were centrifuged at 2,500*g* for 10 min at 4°C. According to the manufacturer’s protocols, we used enzymatic analysis kit (C009-2 and C010-2; Nanjing Jiancheng Bioengineering Institute, Jiangsu, China) to test the activities of glutamic oxaloacetic transaminase (GOT) and glutamic pyruvic transaminase (GPT).

### Quantitative Proteomic Analysis

**Figure 1:**
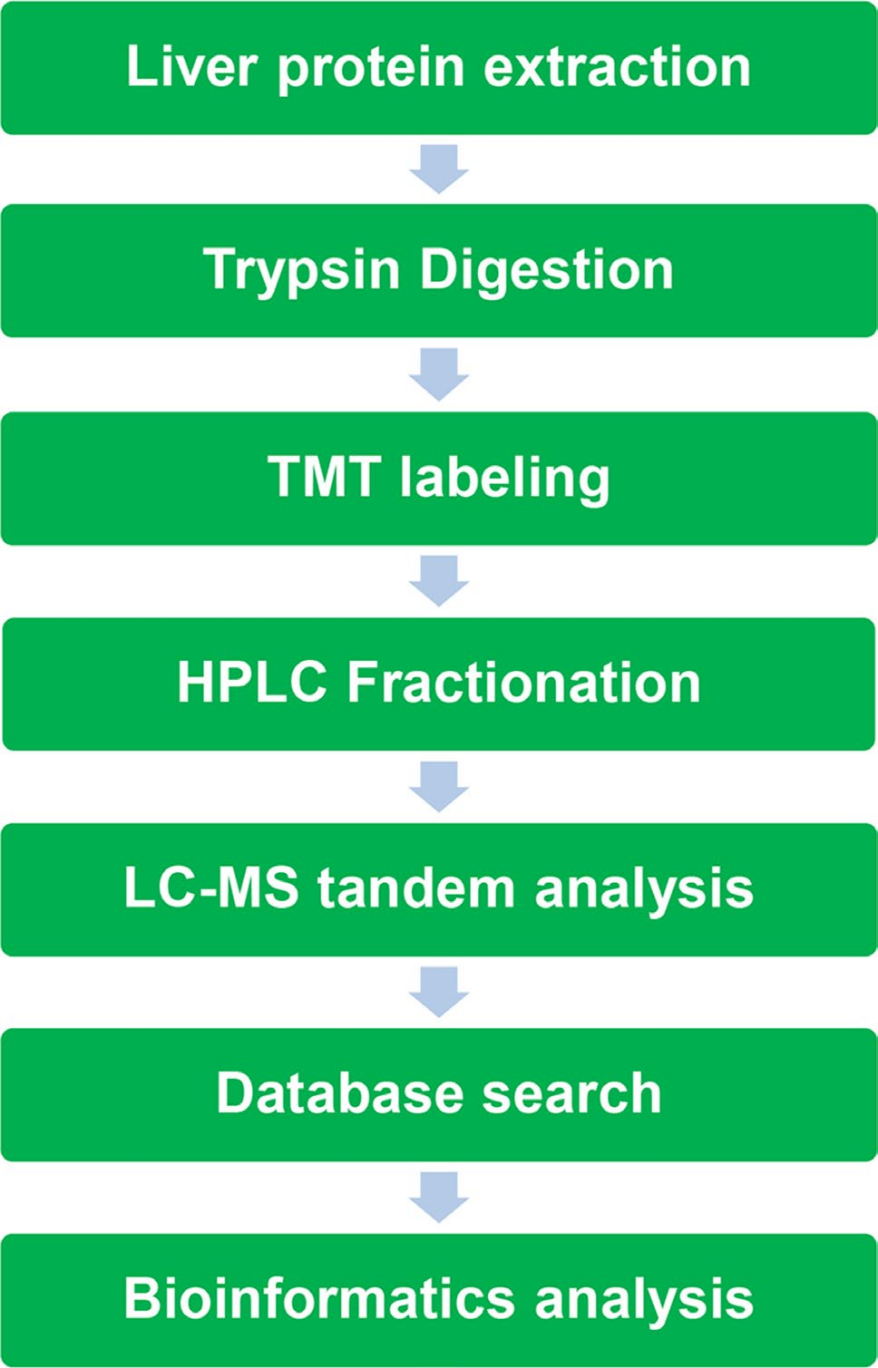
Experimental strategy for quantification of histone lysine acetylation and protein expression in acute liver failure (ALF) mice upon ACY-1215 treatment.

In this project, a series of frontier technologies, such as tandem mass tag (TMT) labeling, high-performance liquid chromatography (HPLC), mass spectrometry-based quantitative proteomics (MS-based quantitative proteomics), and affinity enrichment of histone lysine acetylated peptides were organically combined to study the quantitative proteomics of mouse liver (**Figure 1**).

### Protein–Protein Interaction Network

All differentially expressed protein name identifiers of Model group (M) vs Normal group (N) or ACY-1215 group (A) vs Model group (M) group were searched against the STRING database version 10.5 for protein–protein interactions. Only interactions between the proteins belonging to the searched data set were selected, thereby excluding external candidates. STRING defines a metric called “confidence score” to define interaction confidence. We fetched all interactions that had a confidence score ≥ 0.9 (highest confidence). Interaction network form STRING was visualized in Cytoscape. A graph theoretical clustering algorithm, molecular complex detection (MCODE), was utilized to analyze densely connected regions. MCODE is part of the plug-in tool kit of the network analysis and visualization software Cytoscape.

### Transmission Electron Microscopy

The cells were fixed in 2.5% glutaraldehyde, embedded in epoxy resin, sliced into ultrathin sections (60 nm thicknesses), and stained with 2% uranyl acetate and Reynolds lead citrate. The sections were examined under a HITACHI HT7700 TEM by an electron microscopy specialist from the Department of Ultrastructural Pathology Center, Renmin Hospital of Wuhan University.

### The Detection of Reactive Oxygen Species

The production of ROS was assessed in liver tissues by using the oxidant-sensitive probe 2′,7′-dichlorofluorescein diacetate (DCFH-DA). To summarize, the liver tissues were harvested after the animals were executed and washed with PBS. The frozen sections of liver tissues were stained with DCFH-DA, and the fluorescence of DCF was observed with a fluorescence microscope.

### Western Blotting

All protein samples were extracted from liver tissues in this study, and the samples have been measured by using the bradford protein assays (BCA)-Kit (Thermo, 23227). Fifty milligrams of protein samples was used for sodium dodecyl sulfate (SDS) / polyacrylamide gel electrophoresis (PAGE), and then transferred onto a polyvinylidene difluoride membrane (Millipore, IPFL00010). After blocking with 5% non-fat milk for 1 h, the membranes were incubated overnight at 4°C with the following primary antibodies: Bcl-2 (ab182858), Bax (ab32503) (Abcam, Cambridge, MA, USA) and glyceraldehyde-3-phosphate dehydrogenase (GAPDH) (AB-P-R 001) (Hangzhou Goodhere Biotechnology Co., Ltd, Hanzhou, Zhejiang, China); H2AK5-ac (PTM-106), H2AK9-ac (PTM-125), H2A (PTM-1008), H2BK11-ac (PTM-130), H2BK24-ac (PTM-126), and H2B (PTM-1007) (PTMBiolabs, Inc. Hanzhou, Zhejiang, China). After incubation with secondary antibodies, the specific bands were visualized by an electrochemiluminescence (ECL) detection system.

### Statistical Analysis

Data are expressed as mean ± SD. Differences among groups were determined by two-way ANOVA followed by a *post hoc* Tukey test. Comparisons between two groups were performed by using an unpaired Student’s *t* test. All data were analyzed by statistical product and service solutions (SPSS) 17.0 and *P* < 0.05 was considered to be statistically significant.

## Results

### ACY-1215 Suppressed Necrosis of Hepatocytes and the Improved the Impaired Liver Function in Acute Liver Failure Mice

**Figure 2:**
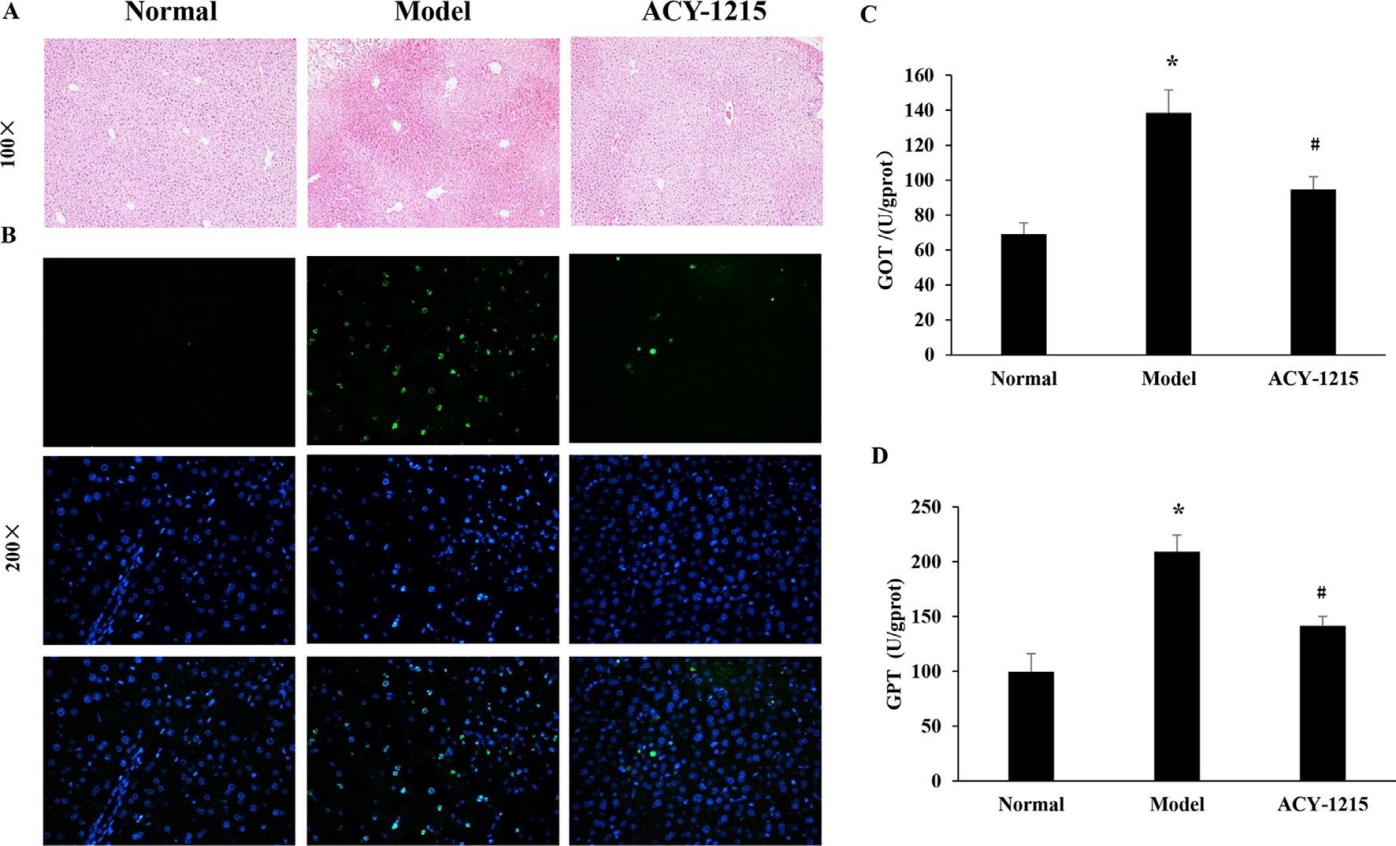
The effect of ACY-1215 treatment of the necrosis of hepatocytes and the impaired liver function in ALF mice. **(A)** The results of the hematoxylin and eosin (HE) staining (original magnification ×100) showed that ACY-1215 could alleviate necrotic changes and preserve the architecture of liver. **(B)** The results of terminal deoxynucleotidyl transferase (TdT)-mediated 2'-Deoxyuridine 5'-Triphosphate (dUTP) Nick-End Labeling (TUNEL) showed the effect of ACY-1215 on the necrosis of hepatocytes. **(C)** Treatment with ACY-1215 normalized the activities of glutamic oxaloacetic transaminase (GOT). **(D)** Treatment with ACY-1215 normalized the activities of glutamate pyruvic transaminase (GPT). *P < 0.05 as compared with the corresponding normal group. ^#^ *P* < 0.05 vs model group.

Compared with the normal group, the normal structure was ruined, and massive necrosis could be observed in ALF group. However, pretreatment with ACY-1215 could remission the cellular necrosis and the hepatic cells redistributed in a radial pattern, which were confirmed by HE staining (**Figure 2A**). Moreover, the result of TUNEL staining demonstrated that the necrosis of hepatocytes in ALF mice was suppressed by ACY-1215 (**Figure 2B**). The levels of GOT and GPT were used to value liver function. The levels of ALT and AST were normalized by treatment with ACY-1215 compared with the ALF mice (**Figure 2C and D**), which indicated that the impaired liver function was improved.

### Impacts of ACY-1215 Treatment on Global Proteome Level in Acute Liver Failure Mice

**Figure 3:**
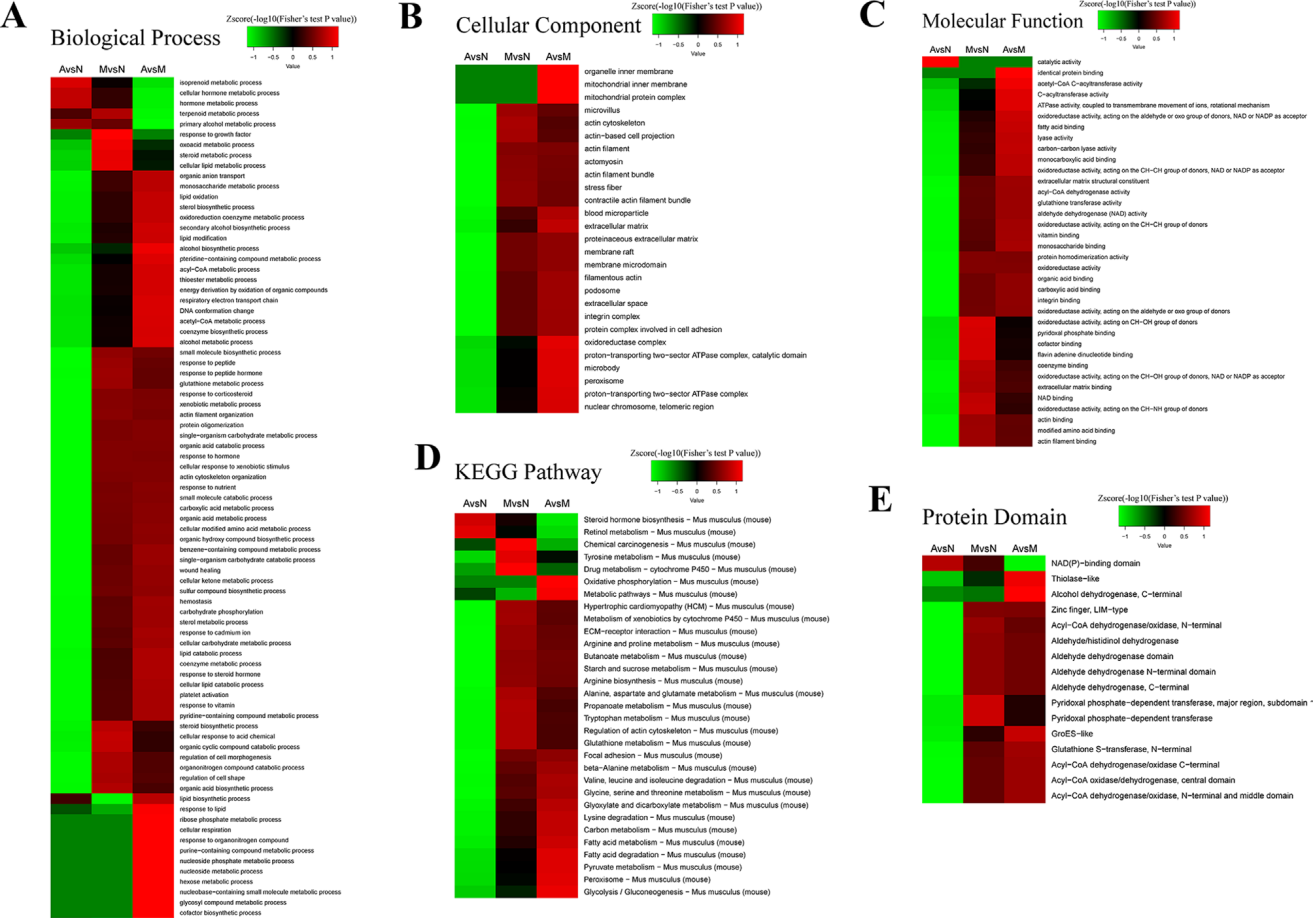
Enrichment and clustering analysis of the quantitative proteomics data sets based on gene ontology (GO) annotations. Quantifiable proteins were classified by gene ontology annotation based on three categories: **(A)** biological process; **(B)** cellular compartment, **(C)** molecular function, **(D)** the kyoto encyclopedia of genes and genomes (KEGG) pathway, and **(E)** the protein domain.

In this project, 4,571 proteins were identified; of which 4,055 have quantitative information. With the threshold change fold >1.5, ACY-1215 treatment induced 1,062 diﬀerentially expressed proteins (470 up-regulated and 592 down-regulated). To reveal the nature of the diﬀerentially expressed proteins upon ACY-1215 treatment, the gene ontology (GO) enrichment-based clustering analysis was carried out. The clustering results of biological process, the cellular component, the molecular function, the KEGG pathway, and the protein domain were shown in **Figure 3A–E**.

For the cellular component analysis (**Figure 3B**), we found that the levels of organelle inner membrane, mitochondrial inner membrane, and mitochondrial protein complex could be normalized by the pretreatment of ACY-1215 in ALF mice, that indicates ACY-1215 may possess a protective role by affecting mitochondrial function. From KEGG pathway (**Figure 3D**), we found that the level of oxidative phosphorylation in ALF mice was increased when compared with normal mice, and pretreatment of ACY-1215 could normalize this trend.

### Protein–Protein Interaction Analysis Reveals the Impacts of ACY-1215 Treatment in Oxidative Phosphorylation in Acute Liver Failure Mice

**Figure 4:**
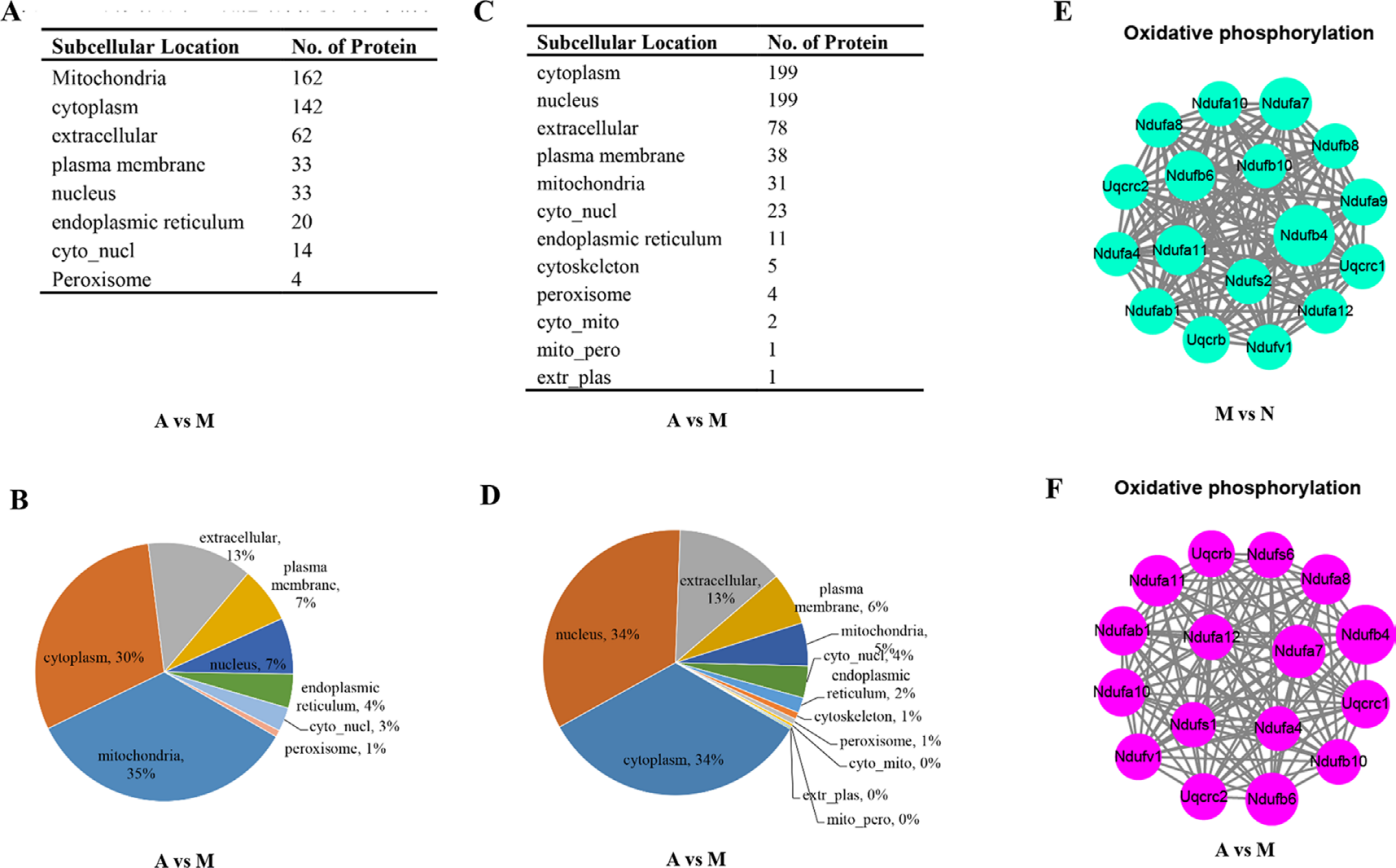
The impacts of ACY-1215 treatment in oxidative phosphorylation in ALF mice. **(A)** and **(B)** Compared with the ALF mice, the subcellular localization and distribution of the up-regulated proteins in ACY-1215 treatment mice. **(C)** and **(D)** Compared with the ALF mice, the subcellular localization and distribution of the down-regulated proteins in ACY-1215 treatment mice. **(E)** Compared with the normal mice, the protein–protein interaction network analyses for the down-regulated oxidative phosphorylation proteins in ALF mice. **(F)** Compared with the ALF mice, the protein–protein interaction network analyses for the up-regulated oxidative phosphorylation proteins in ALF mice with treatment of ACY-1215.

From **Figure 4A and B**, we found that nearly 35% (162 of the 470) up-regulated proteins were located on mitochondria, and there were only 31 of the 592 down-regulated proteins located on mitochondria (**Figure 4C–D**). With the result of **Figure 3B and D**, we then conducted the protein–protein interaction network analyses for the diﬀerent expressed oxidative phosphorylation proteins upon ALF (**Figure 4E**) and ACY-1215-treated mice (**Figure 4F**). The protein–protein interaction network analyses showed that ACY-1215 could normalize the function of electron transport chain, mainly complex I in ALF mice.

### The Effects of ACY-1215 on the Mitochondria-Dependent Reactive Oxygen Species of Apoptosis

**Figure 5:**
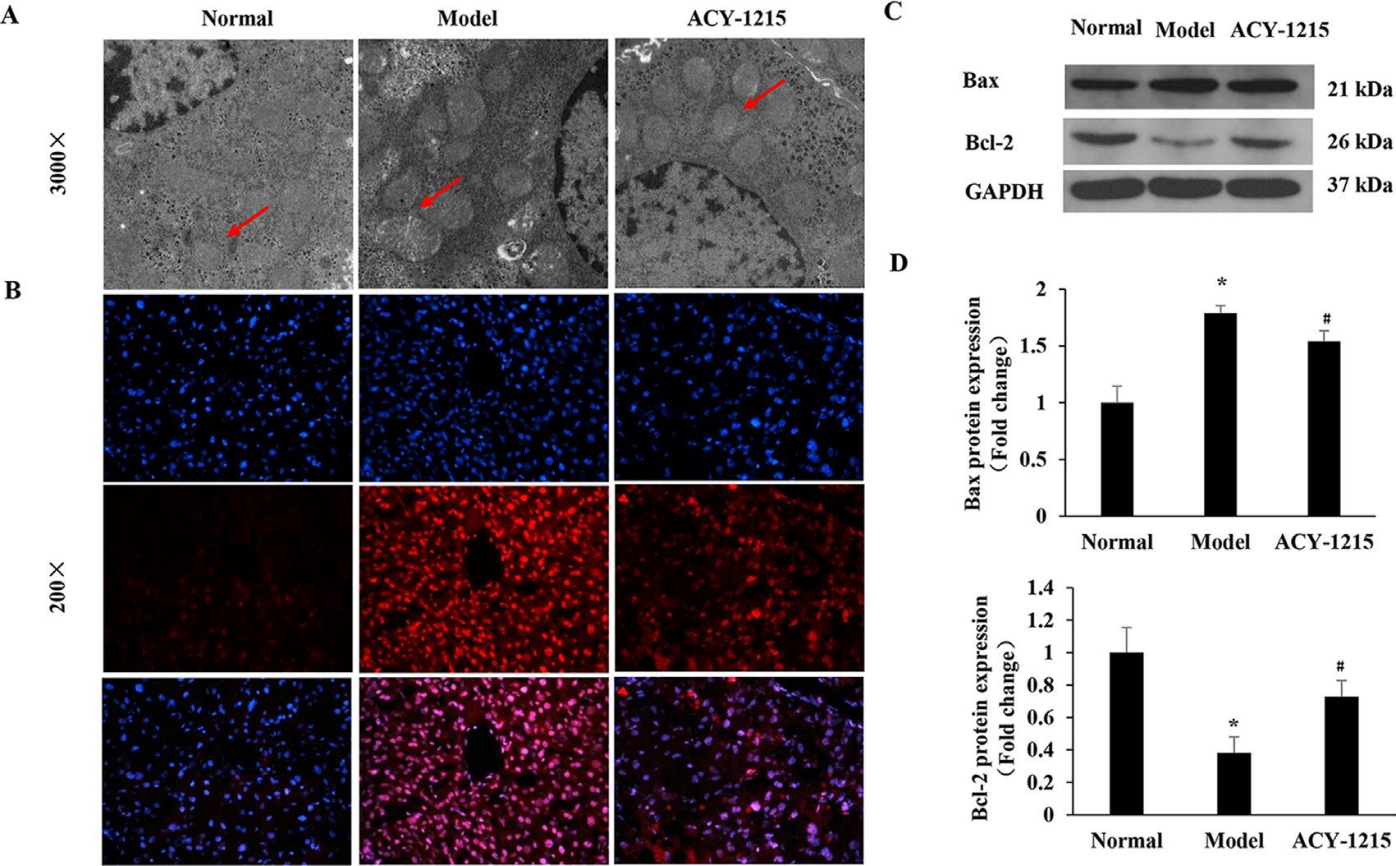
The effects of ACY-1215 on the mitochondria-dependent reactive oxygen species (ROS) of apoptosis. **(A)** Representative transmission electron microscopy TEM images of macrophages on indicated groups. **(B)** The ROS generation in liver tissues was detected by using 2′,7′-dichlorofluorescein diacetate (DCFH-DA). **(C)** The representative expression of Bax and Bcl-2 were analyzed by western blotting. **(D)** The quantitative blots of Bax and Bcl-2 in the indicated groups. *P < 0.05 as compared with the corresponding normal group. ^#^ *P* < 0.05 vs model group.

As shown in **Figure 5A**, compared with the normal group, the ultrastructure of mitochondria in ALF mice were vacuolated and swollen when stimulated with LPS and D-Gal. However, the ultrastructure of mitochondria was significantly improved when the ALF mice were pretreated by ACY-1215. Similarly, treatment of cells with ACY-1215 significantly inhibited ROS generation induced in ALF mice (**Figure 5B**). Moreover, compared with the model group, ACY-1215 could normalize the protein expression levels of Bax and Bcl-2 (**Figure 5C and D**).

### ACY-1215 Treatment Differentially Changed the Lysine Acetylation Level in Histones

The sequences of identified lysine acetylated peptides in core histones and all the corresponding quantitative Kac profiles in response to ACY-1215 treatment were summarized in **Table 1**. The results showed that 94 Kac sites were identified from histones including various histone isoforms, among which 82 sites were quantified (73 sites in Histone 2-4). To reveal that the quantifiable histone lysine acetylation level was really due to ACY-1215 impacts but not histone expression changes, the quantifiable histone acetylation peptides were further normalized according to the quantifiable core histones expression. As indicated, the acetylation level in some sites were significantly increased in response to ACY-1215 treatment, such as H2A.JK6ac, H2A.JK10ac, H2B type 1-AK12ac, and H2B type 1-AK25ac. The acetylation level in some sites showed little changes, including H4K6ac, H4K9ac, and H4K13ac; unexpectedly, we even observed that the acetylation level in some sites were markedly decreased toward ACY-1215 treatment, including H4K80ac and H3.3K57ac.

**Table 1:** Summary of identified Kac sites and quantifiable changes in acute liver function (ALF) mice in response to ACY-1215 treatment.

Kac quantification normalized with proteome				Kac quantification without normalization		Kac quantification normalized with proteome	
Modified sites	Position	Amino acid	Modified sequence	M/N ratio	A/M ratio	M/N ratio	A/M ratio
Histone H2A.J	6	K	GK(1)QGGK(1)VR	0.225	5.103	0.154	7.280
Histone H2A.J	10	K	GK(1)QGGK(1)VR	0.225	5.103	0.154	7.280
Histone H2A.V	8	K	AGK(1)DSGK(1)AK	0.309	4.128	0.212	5.889
Histone H2A.Z	8	K	AGK(1)DSGK(1)AK(1)TK	0.317	4.021	0.217	5.737
Histone H2A.V	5	K	AGGK(1)AGK(1)DSGK(1)AK(1)AK	0.341	4.008	0.234	5.718
Histone H2A.Z	5	K	AGGK(1)AGK(1)DSGK(1)AK(1)TK	0.349	3.889	0.239	5.548
Histone H2A.V	12	K	DSGK(1)AK(1)AK	0.308	3.745	0.211	5.342
Histone H2A.Z	12	K	AGK(1)DSGK(1)AK(1)TK	0.321	3.593	0.220	5.126
Histone H2A type 2-A	6	K	GK(1)QGGK(1)AR	0.451	2.638	0.309	3.763
Histone H2A type 2-A	10	K	GK(1)QGGK(1)AR	0.451	2.638	0.309	3.763
Histone H2A.Z	14	K	AGK(1)DSGK(1)AK(1)TK	0.521	2.193	0.357	3.128
Histone H2B type 1-A	22	K	K(1)GFK(1)K(1)AVTK(1)TQK	0.806	2.238	0.519	2.160
Histone H2B type 1-A	25	K	AVTK(1)TQK(1)K	0.728	2.154	0.469	2.079
Histone H2A type 2-A	96	K	NDEELNK(1)LLGK	1.285	1.397	0.880	1.994
Histone H2B type 2-E	12	K	SAPAPK(1)K(1)GSK(1)K	0.646	1.705	0.417	1.645
Histone H2B type 1-C/E/G	6	K	PEPAK(1)SAPAPK(1)K(1)GSK(1)K	0.755	1.654	0.487	1.596
Histone H2B type 2-B	12	K	SAPAPK(1)K(1)GSK(1)K	0.703	1.630	0.453	1.573
Histone H4	78	K	DAVTYTEHAK(1)R	1.234	1.663	1.254	1.523
Histone H2B type 1-C/E/G	12	K	SAPAPK(1)K(1)GSK(1)K	0.752	1.571	0.485	1.517
Histone H2B type 1-B	12	K	SAPAPK(1)K(1)GSK(1)K	0.739	1.521	0.477	1.468
Histone H4	92	K	TVTAMDVVYALK(1)R	1.908	1.599	1.940	1.464
Histone H2B type 2-B	6	K	PDPAK(1)SAPAPK(1)K(1)GSK(1)K	1.041	1.351	0.671	1.303
Histone H2B type 1-C/E/G	109	K	LLLPGELAK(1)HAVSEGTK	1.096	1.332	0.706	1.285
Histone H2B type 1-H	13	K	SAPAPK(1)K(1)GSK(1)K	0.892	1.320	0.575	1.274
Histone H4	17	K	GLGK(1)GGAK(1)R	0.760	1.363	0.773	1.248
Histone H2B type 1-H	16	K	SAPAPK(1)K(1)GSK(1)K	0.922	1.260	0.595	1.216
Histone H2B type 1-C/E/G	24	K	KAVTK(1)AQK(1)K	0.978	1.224	0.630	1.181
Histone H3.3	28	K	K(1)SAPSTGGVK(0.961)K(0.039)PHR	0.706	1.943	0.831	1.165
Histone H4	9	K	GK(1)GGK(1)GLGK(1)GGAK(1)R	0.932	1.244	0.948	1.139
Histone H4	13	K	GLGK(1)GGAK(1)R	0.860	1.200	0.875	1.099
Histone H3.3	15	K	STGGK(1)APR	0.544	1.817	0.641	1.089
Histone H2B type 1-C/E/G	86	K	LAHYNK(1)R	1.531	1.123	0.987	1.084
Histone H3.3	10	K	K(1)STGGK(1)APR	0.539	1.800	0.635	1.079
Histone H2A type 1-H	96	K	NDEELNK(1)LLGR	2.238	0.749	1.533	1.068
Histone H2B type 1-H	21	K	K(1)GSK(1)K(1)ALTK(1)AQK	1.191	1.104	0.768	1.065
Histone H4	80	K	K(1)TVTAMDVVYALK	2.451	1.162	2.492	1.065
Histone H2B type 1-H	17	K	K(1)GSK(1)K(1)ALTK(1)AQK	1.197	1.073	0.772	1.035
Histone H3.3	38	K	K(1)SAPSTGGVK(0.433)K(0.567)PHR	0.877	1.697	1.032	1.017
Histone H3.3	37	K	K(1)SAPSTGGVK(1)K	0.806	1.686	0.950	1.011
Histone H4	6	K	GK(1)GGK(1)GLGK(1)GGAK(1)R	1.079	1.056	1.097	0.967
Histone H4	32	K	DNIQGITK(1)PAIR	1.367	1.051	1.390	0.962
Histone H2B type 1-B	6	K	PEPSK(1)SAPAPK(1)K(1)GSK	1.333	0.954	0.860	0.921
Histone H2B type 1-C/E/G	21	K	KGSK(1)K(1)AVTK(1)AQK	1.239	0.901	0.799	0.869
Histone H2AX	6	K	GK(1)TGGK(1)AR	0.439	2.689	0.707	0.845
Histone H2AX	10	K	GK(1)TGGK(1)AR	0.439	2.689	0.707	0.845
Histone H2B type 2-B	21	K	K(1)GSK(1)K(1)AVTK(1)VQK	1.118	0.837	0.721	0.808
Histone H2B type 2-B	24	K	KAVTK(1)VQK(1)K	1.248	0.833	0.804	0.804
Histone H3.3	57	K	YQK(1)STELLIR	1.619	1.340	1.907	0.803
Histone H2B type 1-B	13	K	SAPAPK(1)K(1)GSK(1)K	1.281	0.820	0.826	0.792
Histone H2B type 1-B	16	K	SAPAPK(1)K(1)GSK(1)K	1.278	0.811	0.824	0.783
Histone H1.5	63	K	GGVSLPALK(1)K	2.058	0.710	2.355	0.776
Histone H2B type 1-P	12	K	SVPAPK(1)K(1)GSK(1)K	1.351	0.785	0.871	0.758
Histone H2B type 2-B	13	K	SAPAPK(1)K(1)GSK(1)K	1.316	0.783	0.849	0.755
Histone H3.3	80	K	EIAQDFK(1)TDLR	1.282	1.253	1.510	0.751
Histone H2B type 1-B	24	K	K(1)AISK(1)AQK(1)K	1.493	0.770	0.962	0.744
Histone H2B type 1-B	17	K	K(1)GSK(1)K(1)AISK(1)AQK	1.420	0.750	0.915	0.724
Histone H2B type 1-C/E/G	17	K	K(1)GSK(1)K(1)AVTK(1)AQK	1.463	0.747	0.943	0.721
Histone H2B type 1-P	17	K	K(1)GSK(1)K(1)AVTK(1)AQK	1.463	0.747	0.943	0.721
Histone H2B type 2-E	17	K	K(1)GSK(1)K(1)AVTK(1)AQK	1.463	0.747	0.943	0.721
Histone H2B type 1-B	21	K	K(1)GSK(1)K(1)AISK(1)AQK	1.561	0.733	1.007	0.708
Histone H2B type 2-B	16	K	SAPAPK(1)K(1)GSK(1)K	1.390	0.732	0.896	0.706
Histone H2B type 1-C/E/G	13	K	SAPAPK(1)K(1)GSK(1)K	1.484	0.730	0.957	0.705
Histone H3.3	123	K	VTIMPK(1)DIQLAR	1.248	1.163	1.469	0.697
Histone H2B type 1-C/E/G	16	K	SAPAPK(1)K(1)GSK(1)K	1.532	0.687	0.988	0.663
Histone H3.3	19	K	K(1)QLATK(1)AAR	0.978	1.031	1.152	0.618
Histone H2B type 2-E	16	K	SAPAPK(1)K(1)GSK(1)K	1.617	0.633	1.043	0.611
Histone H2B type 2-E	13	K	SAPAPK(1)K(1)GSK(1)K	1.678	0.617	1.082	0.595
Histone H2B type 2-B	17	K	K(1)GSK(1)K(1)AVTK(1)VQK	1.648	0.616	1.063	0.595
Histone H2B type 1-P	16	K	SVPAPK(1)K(1)GSK(1)K	1.765	0.577	1.138	0.557
Histone H2B type 1-P	13	K	SVPAPK(1)K(1)GSK(1)K	1.854	0.556	1.195	0.536
Histone H3.3	24	K	QLATK(1)AAR	1.150	0.857	1.355	0.514
Histone H3.2	37	K	K(1)SAPATGGVK(0.997)K(0.003)PHR	2.245	0.390	1.546	0.412
Histone H3.2	28	K	K(1)SAPATGGVK(0.997)K(0.003)PHR	2.741	0.350	1.887	0.369

Model group (M)/Normal group (N) Ratio, ACY-1215 group (A)/Model group (M) Ratio.

**Figure 6:**
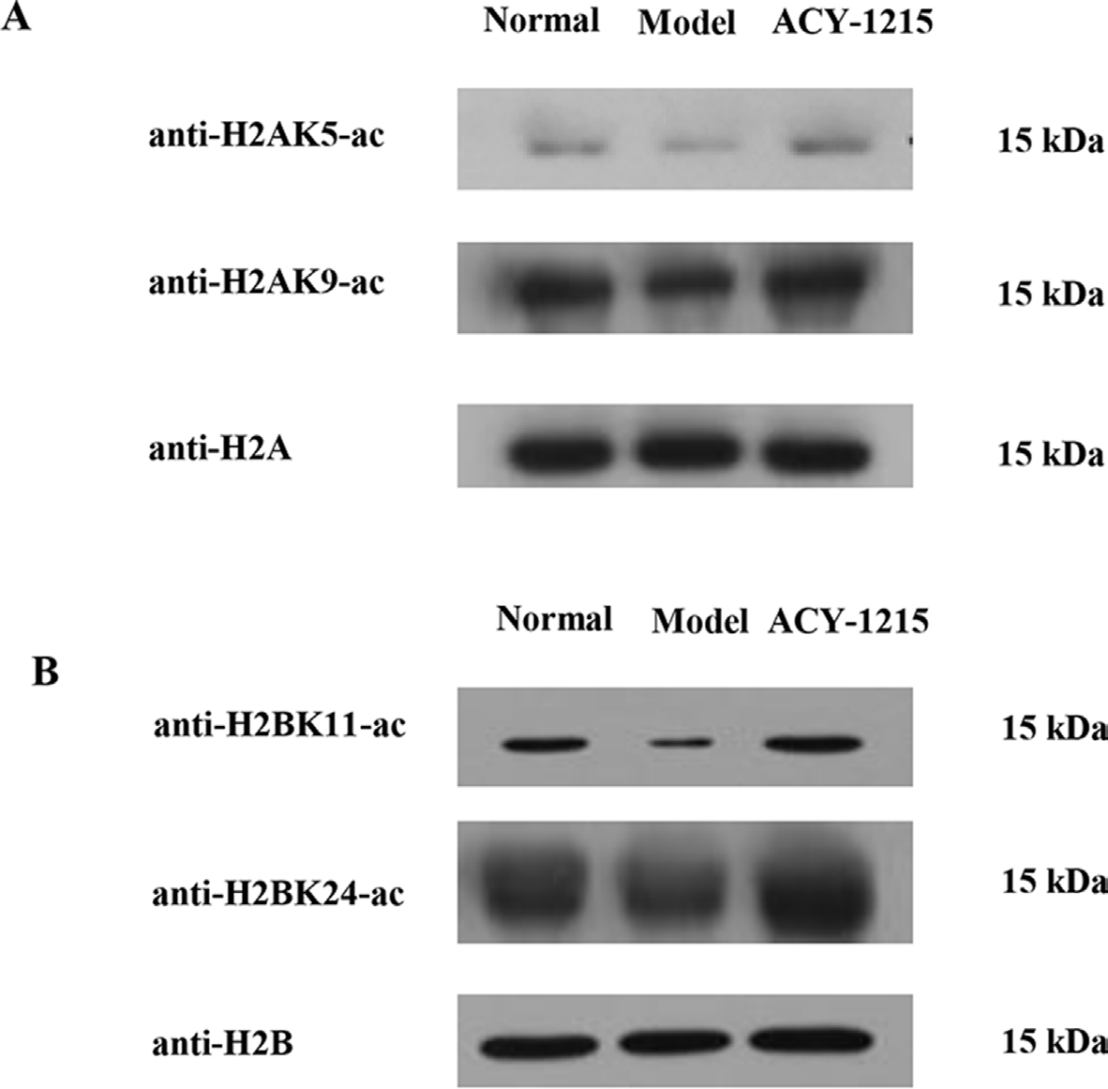
ACY-1215 treatment changed the histone lysine acetylation profiling. The extracted core histones was subject to SDS/PAGE followed by western blotting analysis to exam core histone site-specific lysine acetylation changes in H2A **(A)** and H2B **(B)** using indicated histone site-specific lysine acetylation antibodies.

To further validate the different histone acetylation profiles in response to ACY-1215 treatment revealed by quantitative proteomics analysis, we next performed Western blot analysis with some histone lysine acetylation sequence-specific antibodies. The first amino acid of all proteins is methionine (Met), and when naming the sites of antibody, the Met is usually excluded, that is why the number of sites in antibody are usually less 1 when compared with the sites in the analysis of quantifiable histone acetylation peptides. Because of the similarity of protein subtypes, the antibodies cannot be distinguished the subtypes. We selected several sites with Model group (M)/Normal group (N) Ratio<0.66 and ACY-1215 group (A)/Model group (M) Ratio >1.5 and used antibody H2AK5-ac, H2AK9-ac, H2BK11-ac, and H2BK24-ac to verify the acetylation level of the sites. Consistent with quantitative results summarized in **Table 1**, ACY-1215 treatment significantly increased the acetylation level in H2AK5-ac, H2AK9-ac (**Figure 6A**), H2BK11-ac, and H2BK24-ac (**Figure 6B**). Further studies may focus on those markedly altered lysine acetylated sites in core histones to explore the novel epigenetic mechanism of ACY-1215-mediated ALF therapy.

## Discussion

ALF is a severe clinical syndrome characterized by massive necrosis and inflammation, and there is no effective treatment except liver transplantation for this disease with high mortality (Canbay et al., 2011). Therefore, it is urgent to find new therapeutic drugs and therapeutic targets for ALF. Based on homology to yeast proteins, the 11 zinc-dependent HDACs in humans have been classified as: class I, HDACs 1, 2, 3, and 8; class IIa includes HDACs 4, 5, 7 and 9; class IIb includes HDACs 6 and 10; and class IV HDAC11 (Marks and Xu, 2009). Compared with other zinc-dependent HDACs, HDAC6 is a structurally and functionally unique and exerts its activity by selectively deacetylating multiple target molecules (Namdar et al., 2010; Li et al., 2013). The aberrant expression of HDAC6 may implicate in the progression of various pathological disorders, including inflammatory diseases (Tang et al., 2018). In this study, the hepatic necrosis in ALF models was induced by LPS and D-galN. The results showed that compared with the ALF mice, treatment of ACY-1215 had protective effect in liver structure, liver function, and necrosis of hepatocytes.

Previous studies have shown the protective effect of ACY-1215 in some inflammatory diseases, including ALF (Tang et al., 2018; Zhang et al., 2018b; Cheng et al., 2019), but the proteomic studies of the therapeutically role of ACY-1215 in ALF were still rare. In the present study, we found that with the threshold change fold >1.5, ACY-1215 treatment induced 1062 diﬀerentially expressed proteins (470 up-regulated and 592 down-regulated). Compared with the model group, nearly 35% (162 of the 470) up-regulated proteins were located on mitochondria, however, and there were only 31 of the 592 down-regulated proteins located on mitochondria. As shown in the cellular component category, the mitochondrial inner membrane and mitochondrial protein complex in ALF mice were significantly decreased, and ACY-1215 treatment could reverse that trend. Moreover, as **Figure 3E** has shown, we can see that pretreatment of ACY-1215 could normalize the oxidative phosphorylation in ALF mice, and protein–protein interaction network analyses showed that mainly by normalizing the activity of mitochondrial electron transport chain complex I. Complex I is critical for ROS production by the respiratory chain, and under conditions of inhibition of respiratory complex I, it can generate significant amounts of ROS (Babot et al., 2014; Sumegi et al., 2017). And excess production of ROS can induce cell death by oxidative damaging (Nishi et al., 2017). As a first-in-class selective HDAC6 inhibitor, ACY-1215 has been reported to have the effect to regulate cell apoptosis in tumor (Santo et al., 2012; Cao et al., 2018; Wang et al., 2018). However, there are still rare studies of the effect and mechanism of HDAC6 inhibitor in the necrosis of hepatocytes in ALF. The results of detection of ROS and TEM showed that ACY-1215 could not only normalize the ultrastructure of mitochondria but also decrease the generation of ROS in ALF mice. The protein expression levels of Bax and Bcl-2 were normalized by ACY-1215, which reflected that ACY-1215 treatment could alleviate necrosis of hepatocytes. All results suggested that inhibition of HDAC6 activity by using ACY-1215 could suppress the necrosis of hepatocytes in ALF mice by inhibiting the production of mitochondria-dependent ROS.

Previous studies have reported that HDAC6 could selectively deacetylate non-histone proteins, such as tubulin, HSP90, cortactin, and peroxiredoxin (Parmigiani et al., 2008; Youn et al., 2016; Messaoudi et al., 2017). However, there are still limited studies about sequences of identified lysine acetylated peptides in core histones in response to ACY-1215 treatment. In our study, 94 Kac sites were identified from histones, including various histone isoforms, among which 82 sites were quantified (73 sites in Histone2-4). As indicated, the acetylation level in some sites was significantly increased in response to ACY-1215 treatment. Furthermore, we next performed Western blot analysis with histone lysine acetylation sequence-specific antibodies, the results showed that the acetylation level of sites in Histone2-4 were quite consistent with the result revealed by mass spectrometer analysis. Further studies may focus on those markedly altered lysine acetylated sites in core histones, particularly those increased lysine acetylated sites, to explore the novel epigenetic mechanism of ACY-1215-mediated ALF therapy.

In conclusion, a high dose of ACY-1215 could ameliorate the necrosis of hepatocytes, and the mechanism may be related to the inhibition of mitochondria-dependent ROS of apoptosis. Taking the advantages of quantitative proteomic analysis, important biological processes and functions related with ACY-1215 were revealed, the lysine-acetylated peptides in core histones were identified. These results therefore expand our current understanding of the underlying mechanism of ACY-1215 roles as HDAC6 inhibitor and how ACY-1215 functions in ALF therapy.

## Data Availability Statement

The raw data supporting the conclusions of this manuscript will be made available by the authors, without undue reservation, to any qualified researcher.

## Ethics Statement

All animal experiments were performed in accordance with the institutional guidelines of the Animal Care and Use Committee of Renmin Hospital of Wuhan University and the Guide for the Care of Laboratory Animals published by the US National Institutes of Health (NIH Publication No. 85-23, revised 1996).

## Author Contributions

All authors listed have made a substantial, direct, and intellectual contribution to the work and approved it for publication.

## Funding

The present study was supported by the National Natural Science Foundation of China (grant no. 81870413).

## Conflict of Interest Statement

The authors declare that the research was conducted in the absence of any commercial or financial relationships that could be construed as a potential conflict of interest.

## Abbreviations

ALF, acute liver failure; D-GalN, D-galactosamine; GOT, glutamic oxaloacetic transaminase; GPT, glutamate pyruvic transaminase; HDAC6, Histone deacetylase 6; LPS, lipopolysaccharide; ROS, reactive oxygen species; SPF, specific pathogen-free; TEM, transmission electron microscopy; TLR4, toll like receptor 4; TNF-α, tumor necrosis factor α; IL-1β, interleukin-1β.

## References

[B1] Babot M. Birch A. Labarbuta P. Galkin A. (2014). Characterisation of the active/de-active transition of mitochondrial complex I. Biochim. Biophys. Acta 1837, 1083–1092. 10.1016/j.bbabio.2014.02.018 24569053 PMC4331042

[B2] Bi X. Wang P. Ma Q. Han L. Wang X. Mu Y. (2017). Anti-inflammatory activities and liver protection of Alisol F and 25-Anhydroalisol F through the inhibition of MAPK, STAT3, and NF-κB activation in vitro and in vivo. Molecules 22, pii: E951. 10.3390/molecules22060951 28594379 PMC6152757

[B3] Canbay A. Tacke F. Hadem J. Trautwein C. Gerken G. Manns M. P. (2011). Acute liver failure: a life-threatening disease. Dtsch. Arztebl. Int. 108, 714–720. 10.3238/arztebl.2011.0714 22114640 PMC3221437

[B4] Cao J. Lv W. Wang L. Xu J. Yuan P. Huang S. (2018). Ricolinostat (ACY-1215) suppresses proliferation and promotes apoptosis in esophageal squamous cell carcinoma *via* miR-30d/PI3K/AKT/mTOR and ERK pathways. Cell Death Dis. 9, 817. 10.1038/s41419-018-0788-2 30050135 PMC6062526

[B5] Cheng C. Shan W. Huang W. Ding Z. Cui G. Liu F. (2019). ACY-1215 exhibits anti-inflammatory and chondroprotective effects in human osteoarthritis chondrocytes *via* inhibition of STAT3 and NF-κB signaling pathways. Biomed. Pharmacother. 109, 2464–2471. 10.1016/j.biopha.2018.11.017 30551507

[B6] Jiang L. Zhang S. Hu H. Yang J. Wang X. Ma Y. (2019). Exosomes derived from human umbilical cord mesenchymal stem cells alleviate acute liver failure by reducing the activity of the NLRP3 inflammasome in macrophages. Biochem. Biophys. Res. Commun. 508, 735–741. 10.1016/j.bbrc.2018.11.189 30528233

[B7] Kim S. J. Lee S. M. (2013). NLRP3 inflammasome activation in D-galactosamine and lipopolysaccharide-induced acute liver failure: role of heme oxygenase-1. Free. Radic. Biol. Med. 65, 997–1004. 10.1016/j.freeradbiomed.2013.08.178 23994575

[B8] Li Y. Shin D. Kwon S. H. (2013). Histone deacetylase 6 plays a role as a distinct regulator of diverse cellular processes. FEBS J. 280, 775–793. 10.1111/febs.12079 23181831

[B9] Marks P. A. Xu W. S. (2009). Histone deacetylase inhibitors: potential in cancer therapy. J. Cell Biochem. 10, 600–608. 10.1002/jcb.22185 PMC2766855 19459166

[B10] Messaoudi K. Ali A. Ishaq R. Palazzo A. Sliwa D. Bluteau O. (2017). Critical role of the HDAC6-cortactin axis in human megakaryocyte maturation leading to a proplatelet-formation defect. Nat. Commun. 8, 1786. 10.1038/s41467-017-01690-2 29176689 PMC5702605

[B11] Namdar M. Perez G. Ngo L. Marks P. A. (2010). Selective inhibition of histone deacetylase 6 (HDAC6) induces DNA damage and sensitizes transformed cells to anticancer agents. Proc. Natl. Acad. Sci. U.S.A. 107, 20003–20008. 10.1073/pnas.1013754107 21037108 PMC2993347

[B12] Nishi K. Iwaihara Y. Tsunoda T. Doi K. Sakata T. Shirasawa S. (2017). ROS-induced cleavage of NHLRC2 by caspase-8 leads to apoptotic cell death in the HCT116 human colon cancer cell line. Cell Death Dis. 8, 3218. 10.1038/s41419-017-0006-7 29242562 PMC5870588

[B13] Parmigiani R. B. Xu W. S. Venta-Perez G. Erdjument-Bromage H. Yaneva M. Tempst P. (2008). HDAC6 is a specific deacetylase of peroxiredoxins and is involved in redox regulation. Proc. Natl. Acad. Sci. U.S.A. 105, 9633–9638. 10.1073/pnas.0803749105 18606987 PMC2443817

[B14] Prince H. M. Bishton M. J. Harrison S. J. (2009). Clinical studies of histone deacetylase inhibitors. Clin. Cancer Res. 15, 3958–3969. 10.1158/1078-0432.CCR-08-2785 19509172

[B15] Rabinowich L. Wendon J. Bernal W. Shibolet O. (2016). Clinical management of acute liver failure: results of an international multi-center survey. World J. Gastroenterol. 22, 7595–7603. 10.3748/wjg.v22.i33.7595 27672280 PMC5011673

[B16] Santo L. Hideshima T. Kung A. L. Tseng J. C. Tamang D. Yang M. (2012). Preclinical activity, pharmacodynamic, and pharmacokinetic properties of a selective HDAC6 inhibitor, ACY-1215, in combination with bortezomib in multiple myeloma. Blood 119, 2579–2589. 10.1182/blood-2011-10-387365 22262760 PMC3337713

[B17] Sumegi K. Fekete K. Antus C. Debreceni B. Hocsak E. Gallyas F. Jr. (2017). BGP-15 protects against oxidative stress- or lipopolysaccharide-induced mitochondrial destabilization and reduces mitochondrial production of reactive oxygen species. PLoS One 12, e0169372. 10.1371/journal.pone.0169372 28046125 PMC5207682

[B18] Tang J. Shi Y. Liu N. Xu L. Zang X. Li P. (2018). Blockade of histone deacetylase 6 protects against cisplatin-induced acute kidney injury. Clin. Sci. (Lond.) 132, 339–359. 10.1042/CS20171417 29358506

[B19] Wang X. X. Wan R. Z. Liu Z. P. (2018). Recent advances in the discovery of potent and selective HDAC6 inhibitors. Eur. J. Med. Chem. 143, 1406–1418. 10.1016/j.ejmech.2017.10.040 29133060

[B20] Wang Y. Wang J. L. Ma H. C. Tang Z. T. Ding H. R. Shi X. L. (2019). Mesenchymal stem cells increase heme oxygenase 1-activated autophagy in treatment of acute liver failure. Biochem. Biophys. Res. Commun. 508, 682–689. 10.1016/j.bbrc.2018.11.146 30528392

[B21] Youn G. S. Lee K. W. Choi S. Y. Park J. (2016). Overexpression of HDAC6 induces pro-inflammatory responses by regulating ROS-MAPK-NF-κB/AP-1 signaling pathways in macrophages. Free Radic. Biol. Med. 97, 14–23. 10.1016/j.freeradbiomed.2016.05.014 27208785

[B22] Zhang Q. Yang F. Li X. Zhang H. Y. Chu X. G. Zhang H. (2015). Trichostatin A protects against experimental acute-on-chronic liver failure in rats through regulating the acetylation of nuclear factor-kappaB. Inflammation 38, 1364–1373. 10.1007/s10753-014-0108-7 25604312

[B23] Zhang Q. Yang F. Li X. Li X. Zhang H. Y. Chu X. G. (2016). Trichostatin A protects against intestinal injury in rats with acute liver failure. J. Surg. Res. 205, 1–10. 10.1016/j.jss.2016.05.028 27620992

[B24] Zhang Y. Li Y. Li W. Cai J. Yue M. Jiang L. (2018a). Therapeutic effect of human umbilical cord mesenchymal stem cells at various passages on acute liver failure in rats. Stem. Cells Int. 2018, 7159465. 10.1155/2018/7159465 30538751 PMC6261392

[B25] Zhang H. Zhang W. Jiao F. Li X. Zhang H. Wang L. (2018b). The nephroprotective effect of MS-275 on lipopolysaccharide (LPS)-induced acute kidney injury by inhibiting reactive oxygen species (ROS)-oxidative stress and endoplasmic reticulum stress. Med. Sci. Monit. 24, 2620–2630. 10.12659/MSM.906362 29704392 PMC5944402

[B26] Zhang W. B. Zhang H. Y. Jiao F. Z. Wang L. W. Zhang H. Gong Z. J. (2018c). Histone deacetylase 6 inhibitor ACY-1215 protects against experimental acute liver failure by regulating the TLR4-MAPK/NF-κB pathway. Biomed. Pharmacother. 97, 818–824. 10.1016/j.biopha.2017.10.103 29112935

[B27] Zong L. Yu Q. H. Du Y. X. Deng X. M. (2014). Edaravone protects endotoxin-induced liver injury by inhibiting apoptosis and reducing proinflammatory cytokines. Braz. J. Med. Biol. Res. 47, 231–236. 10.1590/1414-431X20133186 24554039 PMC3982944

